# Renal aspergillosis after COVID‐19‐associated pulmonary aspergillosis: A case report

**DOI:** 10.1002/ccr3.7882

**Published:** 2023-09-07

**Authors:** Roya Ghasemian, Lale Vahedi Larijani, Kimia Rasouli, Mohammad Taghi Hedayati, Chanour Tavakol, Keyvan Heydari, Hamidreza Zalpoor, Aref Hoseini

**Affiliations:** ^1^ Department of Infectious Disease Antimicrobial Resistance Research Center, Mazandaran University of Medical Sciences Sari Iran; ^2^ Department of Pathology, School of Medicine Mazandaran University of Medical Sciences Sari Iran; ^3^ Gastrointestinal Cancer Research Center, Non‐Communicable Diseases Institute, Mazandaran University of Medical Sciences Sari Iran; ^4^ Student Research Committee, School of Medicine Mazandaran University of Medical Sciences Sari Iran; ^5^ Department of Medical Mycology, School of Medicine Mazandaran University of Medical Sciences Sari Iran; ^6^ Invasive Fungi Research Center/Department of Medical Mycology, School of Medicine Mazandaran University of Medical Sciences Sari Iran; ^7^ Tehran School of Medicine Tehran University of Medical Sciences Tehran Iran; ^8^ Shiraz Neuroscience Research Center, Shiraz University of Medical Sciences Shiraz Iran; ^9^ Network of Immunity in Infection, Malignancy & Autoimmunity (NIIMA), Universal Scientific Education & Research Network (USERN) Tehran Iran; ^10^ American Association of Kidney Patients (AAKP) Tampa Florida USA

**Keywords:** aspergillus flavus, case report, COVID‐19, pulmonary aspergillosis, renal aspergillosis, voriconazole

## Abstract

**Key Clinical Message:**

Renal aspergillosis is a rare condition and this case the first case of Renal aspergillosis reported after COVID‐19‐associated pulmonary aspergillosis. Renal symptoms should arise clinical suspicion to renal involvement that happened as a result of hematogenous spreading of pulmonary aspergillosis.

**Abstract:**

Secondary fungal infections are among the most significant complications that can arise after COVID‐19 and have the potential to lead to a high rate of morbidity and mortality. As COVID‐19 primarily involves the airway, the majority of fungal infections reported have been related to the respiratory system. However, renal aspergillosis that we have reported is a rare condition that also can occur. A 67‐year‐old man was referred to our hospital and admitted as a COVID‐19 patient. After the initial recovery, he experienced a recurrence of fever accompanied by a productive cough. The histopathological studies were conducted on the sputum and bronchoalveolar lavage samples, which revealed the presence of *Aspergillus flavus*. We treated the patient with voriconazole and the patient was discharged after a period of time. However, after approximately 6 months, he returned to the hospital with a fever and abdominal pain. We started a fever workup. Two new hypoechoic abscess‐like masses were spotted in the right kidney in the ultrasonography (U/S) and the direct molecular studies of the biopsy sample obtained under U/S guidance identified *Aspergillus flavus*. Although renal aspergillosis is a rare condition, it should not be overlooked, especially in patients with severe COVID‐19 and pulmonary aspergillosis, as these conditions can lead to renal aspergillosis, which may present with symptoms such as abdominal pain with fever. Therefore, it is necessary to perform radiological and histopathological studies when renal aspergillosis is suspected.

## Graphical abstract

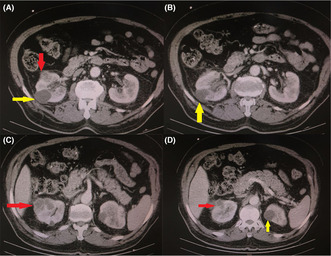

## 1 INTRODUCTION

In 2019, the World Health Organization (WHO) declared a pandemic caused by a novel coronavirus. The resulting global challenge, known as coronavirus disease 2019 (COVID‐19), is primarily characterized by symptoms such as fever, cough, dyspnea, fatigue, and other presentations that may vary in different situations.^1^ This disease has infected millions of people worldwide, with a mortality rate of over 3%. Unlike the beginning of this pandemic, superinfection reports (particularly fungal superinfection reports) have shown an ascending trend.^2^ The role of opportunistic fungal infections in the morbidity and mortality of COVID‐19 patients seems to be significant and decisive. There are numerous reports of fungal infections involving lungs, orofacial, and orbital area in COVID‐19 patients. The COVID‐19 patients exposed to high risk are those with acute respiratory distress syndrome (ARDS) in the intensive care unit (ICU), who receive broad‐spectrum antibiotic agents or immunosuppressant agents and are supported by mechanical ventilation. These patients are more likely to develop COVID‐19‐associated pulmonary aspergillosis (CAPA).^3^, ^4^, ^5^ In addition, danger‐associated molecule release during severe COVID‐19 could predispose the patient to CAPA.^6^ CAPA incidence is estimated to be more than 10% while it has more than 48% of mortality rate in these patients.^7^, ^8^

Primary renal aspergillosis is an uncommon urological entity that mostly occurs in immunocompromised patients.^9^ Renal aspergillosis is mainly because of hematogenous infection spread from lungs secondary to pulmonary aspergillosis, and in terms of the form, it may appear as focal abscesses and fungal bezoars.^10^ We report a case of renal aspergillosis, which is the first case reported after a COVID‐19 episode following COVID‐19‐associated CAPA.

## 2 CASE PRESENTATION

In August 2020, a 69‐year‐old man was referred to our emergency department with a 9‐day history of fever, sweating, cough, and diarrhea. The patient had a history of diabetes mellitus, benign prostatic hyperplasia, ischemic heart disease, spinal tuberculosis, and renal stone in his past medical history. He was taking oral antihyperglycemic agents (including metformin, glibenclamide, and acarbose) for his diabetes, as well as 80 mg of daily aspirin, 40 mg of daily atorvastatin, 25 mg of losartan BID, 25 mg of metoprolol BID and 5 mg of finasteride daily. The patient had axillary 38.6°C temperature, 112 pulse rates per minute, 22 respiratory rates per minute, 135/85 mmHg of blood pressure. Oxygen saturation was 81% and considering the oxygen saturation and patient's clinical condition, noninvasive mechanical ventilation started. Hereupon, the patient underwent drug prescription and para‐clinical collaboration due to clinical suspicion of COVID‐19.

The primary lab data showed leukocytosis with lymphopenia, and the increased level of C‐reactive protein (CRP = 30) and lactate dehydrogenase (LDH = 1022). Spiral lung CT scan revealed 60%–70% of lung involvement with bilateral ground glass opacities and real‐time polymerase chain reaction (RT‐PCR) for COVID‐19 was positive. Therefore, we started treating him with antiviral, antibiotics and anti‐inflammatory agents according to the guidelines and patient's general condition. According to the patient's symptoms and vital signs and the sepsis stage, vancomycin, ciprofloxacin and meropenem were prescribed. The patient was admitted to the ICU and ventilated by noninvasive mechanical ventilation and treated with Remdisivir for 5 consecutive days and dexamethasone as a corticosteroid. The dosage of corticosteroid agent was increased for 4 days and finally was replaced by 125 mg of IV methylprednisolone BID for nine consecutive days in order to control cytokine storm. The above mentioned dosage was tapered gradually to dexamethasone considering the patient's general condition's improvement, the primary symptoms subsiding, oxygen saturation increase and the relative lab data improvement. It is necessary to mention that antihyperglycemic agents are discontinued and insulin is prescribed instead according to the guidelines.

Upon being admitted, after primary recovery, after about a week of being admitted, the patient developed fever and harsh productive coughs. Sputum smear and culture were performed, along with interferon‐gamma release assay (IGRA) test due to the past history of TB, and broad‐spectrum antibiotic agent was prescribed. Any para‐clinical consideration was negative for TB, and on the contrary, *Aspergillus spp* was found in the culture medium. Broncho alveolar lavage (BAL) was performed and the pathology lab findings confirmed *aspergillus spp* regarding the diagnosis, that is, COVID‐associated CAPA. We treated the patient with IV amphotericin B for 11 days plus voriconazole. Afterward, voriconazole was continued based on the treatment guidelines. As the patient's general condition improved and the lab findings were obtained, he was discharged with suitable clinical and lab data findings after 2 months of being admitted in October 2020. We prescribed 200 mg of voriconazole daily for 3 months after discharge and treated him like a patient with neutropenia due to a lack of evidence for continuing the antifungal treatment after CAPA.

It is worth mentioning that an abdominal U/S was done for the patient, due to his LUQ abdominal pain at the time of admission. Finding some small renal stones with maximum size of 5 mm in the middle calyx of his left kidney led us to address the pain of these stones. The size and parenchymal echo were normal on both sides. U/S found a simple cortical cyst of 22 mm in size in the mid portion of the right kidney and another one of 32 mm in size in the superior pole of the left kidney. The above‐mentioned pain was resolved after hydration and administration of analgesic agents within 3 days.

As the patient had no respiratory or general symptoms, he was allowed to stop antifungal agent consumption in January 2021. However, a month later, he experienced fatigue and fluctuating fever that responded to acetaminophen. Over the following 2 months, he developed odynophagia. The endoscopic study revealed candidal esophagitis for which fluconazole was started for the patient for 3 weeks with the dosage of 200 mg, which as a result of that the symptoms subsided.

Consequently, after a couple of weeks, the patient experienced fever again but this time with dysuria and hypogastric pain. The patient was treated with 500 mg of ciprofloxacin for 10 days on suspicion of urinary tract infection (UTI) before returning to our clinic and he had been suffering from recurrent UTI sessions and body temperature fluctuation. Eventually, he was admitted again in July 2021 for a fever source investigation.

About 9 months after being discharged for the first time, he was readmitted with fever and abdominal pain for the second time which were considered in UTI first. He complained of fever, fatigue, dysuria, urgency, and hypogastric pain. In physical examination, he had a 38.8°C and had left costovertebral (CV) angle tenderness. Furthermore, he felt a hypogastric tenderness and there were no other findings in his history and P/E. Para‐clinical studies were going to be performed to discover the source of the fever. The laboratory data are summarized in Table 1.

**TABLE 1:** Para‐clinical findings.

Laboratory parameter	Normal range	On admission
Hgb (g/dL)	12–16	8.7
WBC (×10^3^)	4.5–11	12.4
Plt (×10^3^)	130–400	256
ESR (mm/h)	0–22	115
CRP (mg/dL)	Up to 6	73
Cr (mg/dL)	0.5–1	1.1
Urea (mg/dL)	7–20	40
Urine analysis	WBC: Negative RBC: Negative	WBC: 16–18 RBC: 0–1
Urine culture	Negative	Negative
Blood culture	Negative	Negative

Abbreviations: Cr, creatinine; CRP, C‐reactive protein; ESR, erythrocyte sedimentation rate; Hbg, hemoglobin; RBC, red blood count; WBC, white blood count.

Following the patient's readmission with fever and abdominal pain, a spiral lung CT scan and an abdominal U/S were performed, and this led us to an acceptable reason for the source of fever. Kidneys were observed in abdominal U/S. The size and parenchymal echo were normal on both sides, without any signs of renal stones. Two hypoechoic masses were seen in the middle of right kidney (37 by 39 mm) and the superior pole of the same side (50 by 45 mm). A simple cortical cyst of size 33 mm and another one of size 30 mm were observed at the inferior pole of the right kidney and the inferior pole of the left kidney, respectively. As a result of these findings and the difference between the first admission U/S from the second one, we performed an abdominal CT scan with and without contrast for the patient suspected of renal cell carcinoma.

**FIGURE 1:**
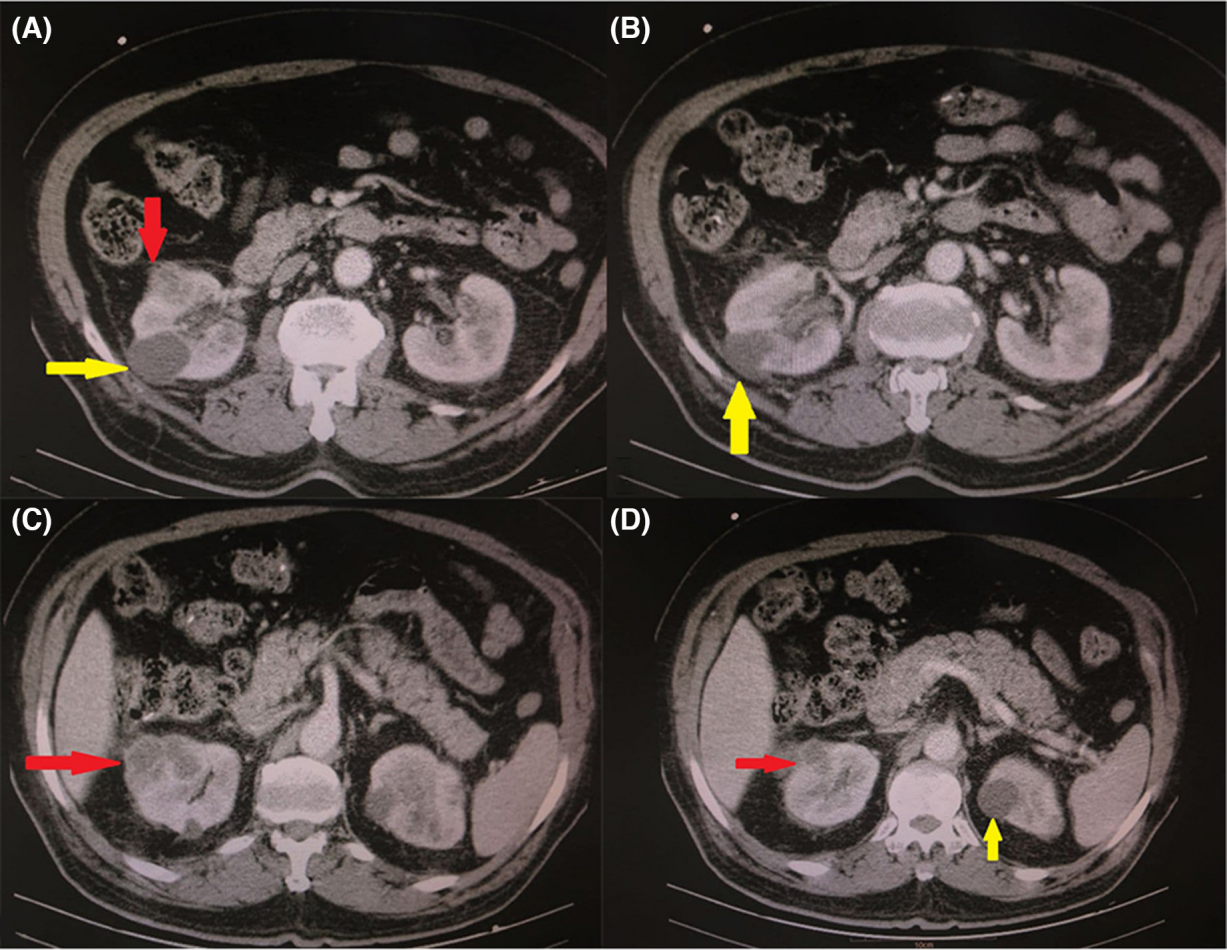
Abdominal CT displays hypodense masses in right kidney (red arrows) and the two simple cysts in both sides (yellow arrows).

**FIGURE 2:**
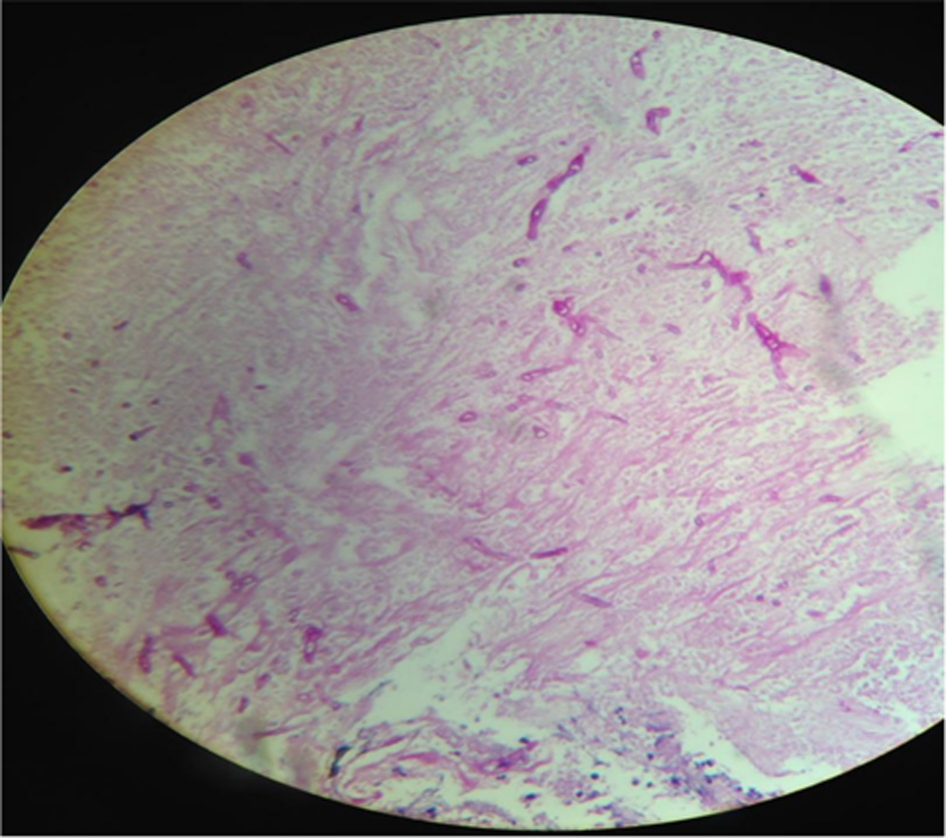
Renal biopsy sample.

Figure 1 depicts two hypoechoic abscess‐like approximately well‐defined round masses in the right kidney. A biopsy was performed under U/S observation from one of the masses (Figure 2) with a trocar needle (14 mm). The pathology lab findings confirmed *Aspergillus. spp* from the smear of the biopsy sample but the sample culture was negative in the following. It is worth noting that using direct molecular study and beta‐tubulin gene sequencing in order to morphologic and molecular recognition of the accurate species revealed *Aspergillus flavus*.

**FIGURE 3:**
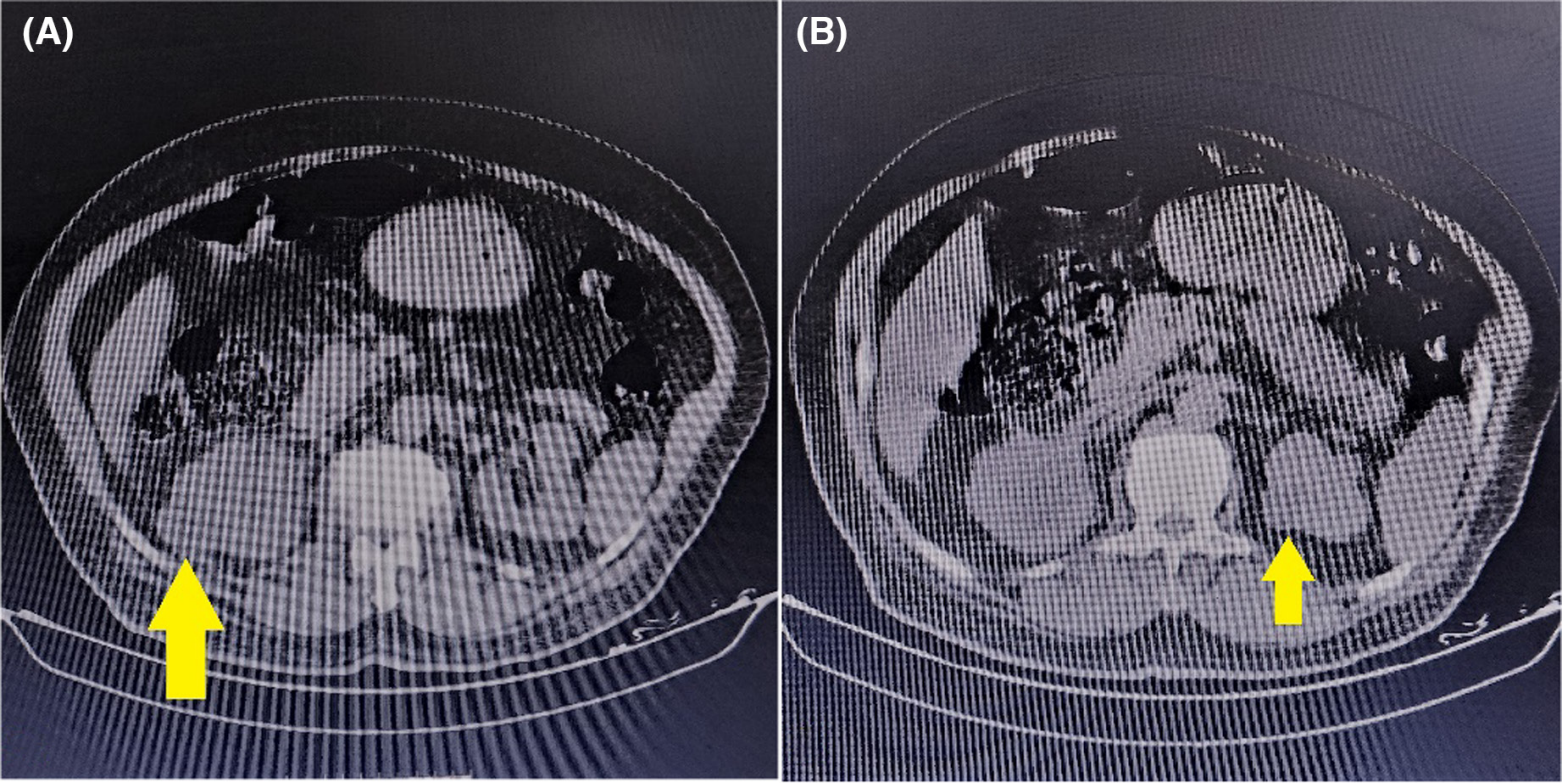
Abdominal CT shows two simple cysts (yellow arrow) in both kidneys and the fungal masses were resolved after a year.

In advance, amphotericin B was prescribed until the susceptibility test results were obtained. However, after 2 days, we had to discontinue it as the patient experienced numbness in his fingers, fatigue and itching, which we attributed to the drug adverse effects and resolved after discontinuing it. Moreover, 200 mg of voriconazole BID was prescribed for the patient, as a result of which, his fever and abdominal pain subsided after 3 days. Susceptibility test result confirmed the susceptibility to voriconazole too. Then, as the patient's general condition improved, he was discharged after 3 weeks of hospitalization and continued voriconazole with above‐mentioned dosage as an outpatient during the next 6 months. The patient underwent an abdominal CT after a year and except the two simple cysts, the masses were resolved (Figure 3).

It is noteworthy that the patient is currently in good health after a year; however, he experiences occasional episodes of mild abdominal pain. Nevertheless, it is important to mention that all of the patient's symptoms have either resolved or dramatically improved.

## 3 DISCUSSION

Secondary fungal infection is one of the most important complications due to COVID‐19. Critically ill patients, those with ARDS, and patients on corticosteroids are more likely to develop CAPA.^11^, ^12^, ^13^ It is necessary to mention that, hypertension and diabetes mellitus are the most common comorbidities in patients with secondary fungal infections in the context of COVID‐19, which was also the case with our patient.^14^, ^15^, ^16^ Considerably, this past medical history has definitely played a role in both episodes of fungal infections. CAPA should be proven by histopathological studies or direct microscopic detection or both. The first line treatment for CAPA is voriconazole or isavuconazole, but liposomal amphotericin B could be considered if epidemiologically drug‐resistant patterns support it before the susceptibility testing is available.^11^

Severe COVID‐19 survivors are exposed to higher risk for kidney involvement during the post‐acute phase of the disease.^17^ COVID‐19 could involve the kidney in different ways. Kidney involvement mostly manifests itself with proteinuria and acute kidney injury (AKI) within 3 weeks after the onset of symptoms.^18^

Renal aspergillosis is an uncommon form of aspergillosis mainly found in immunocompromised patients. Aspergillosis is a primary pulmonary disease, but through their angioinvasive option, aspergillus species could involve extrapulmonary organs by spreading hematogenous from lungs. Renal aspergillosis usually exposes itself by forming abscesses and fungal bezoars may lead to urinary obstruction.^10^, ^19^ It seems that fungi were spread after CAPA involvement in the first admission time and those fungi centers in the kidney made an appearance after stopping the antifungal agent. Corticosteroids and broad‐spectrum antibiotics used for COVID‐19 predisposed the patient to experiencing these fungal infections. Of course, we should assign a share for COVID‐19 itself.

Radiological studies are an inseparable part of diagnosing renal aspergillosis, but the definite diagnosis is made by histopathological and/or microbiological studies based on the biopsy sample. On the other hand, the differential diagnosis, including the primary and metastatic renal malignancies, pyelonephritis, and secondary abscess formation, granulomatous disorders, and renal infarction should be considered.^20^ Better to notice, although the patient does not include the criteria for the autosomal‐dominant polycystic kidney disease (ADPKD), seeing cyst infection in ADPKD patients is a prevalent complication that can result from fungi.^21^, ^22^ Based on the observation and the changes between the first admission U/S and the second one, we decided to biopsy the new masses and consequently, the histopathological findings were as we mentioned above: *Aspergillus flavus*.

## 4 CONCLUSION

COVID‐19‐associated pulmonary aspergillosis (CAPA) was a matter of concern, but it is not where COVID‐19 show finishes. *Aspergillus* spp. could spread hematogenously to extrapulmonary organs, so the patient should be carefully observed. A recurrence of fever should raise clinical suspicion about the recurrence of pulmonary aspergillosis and/or extrapulmonary aspergillosis. Signs, symptoms and laboratory findings must be carefully observed, examined, and interpreted in order to diagnose and initiate timely treatment. This is the first reported case of renal aspergillosis following CAPA. Any urinary tract problems, including urinary tract obstruction, active urine analysis (U/A) and abdominal pain should raise suspicion. Renal aspergillosis is a rare condition, but it should not be overlooked in immunocompromised patients, especially in severe COVID‐19 patients who have suffered from CAPA. Further studies are recommended to recognize and compare the pathophysiology of renal aspergillosis after severe COVID‐19 and CAPA.

## AUTHOR CONTRIBUTIONS

**Roya Ghasemian:** Project administration; resources. **Lale Vahedi Larijani:** Project administration; resources. **Kimia Rasouli:** Project administration; resources. **Mohammad Taghi Hedayati:** Project administration; resources. **Chanour Tavakol:** Project administration; resources. **Keyvan Heydari:** Conceptualization; project administration; resources; writing – original draft; writing – review and editing. **Hamidreza Zalpoor:** Project administration; resources; writing – review and editing. **Aref Hoseini:** Conceptualization; methodology; project administration; resources; supervision; validation; visualization; writing – original draft; writing – review and editing.

## FUNDING INFORMATION

This research did not receive any specific grant from funding agencies in the public, commercial, or not‐for‐profit sectors.

## CONFLICT OF INTEREST STATEMENT

The authors declare no competing interest relevant to the contents of this article.

## ETHICS STATEMENT

The study protocol was approved by the Ethics Committee of Mazandaran University of Medical Sciences.

## CONSENT

Written informed consent was obtained from the patient for publication of this case report and any accompanying images. A copy of the written consent is available for review by the Editor‐in‐Chief of this journal.

## Data Availability

All data generated or analyzed during this study are included in this published article (and its additional information files).
